# Cervical length screening among low-risk women; relationship of body mass index on cervical length and risk of preterm birth

**DOI:** 10.1186/s12884-024-06552-6

**Published:** 2024-05-15

**Authors:** Nurul Iftida Basri, Rima Anggrena Dasrilsyah, Amilia Afzan Mohd Jamil, Charmaine Sook Yee Leong

**Affiliations:** 1 Department of Obstetrics & Gynaecology, Faculty of Medicine & Health Sciences, Universiti Putra Malaysia, Serdang, Selangor 43400 Malaysia; 2 Department of Obstetrics & Gynaecology, Hospital Sultan Abdul Aziz Shah, Universiti Putra Malaysia, Serdang, Selangor Malaysia

**Keywords:** Cervical length, Body mass index, Preterm birth, Premature delivery

## Abstract

**Background:**

Preterm birth (PTB) contributes to nearly 11% of all deliveries in the world. The majority of spontaneous preterm birth (sPTB) remains unexplained. Risk factors include abnormal body mass index (BMI), short cervical length, comorbidities and many more. However, there is limited study on the association between body mass index, cervical length and preterm birth in Malaysia among low-risk women. Hence, we aim to examine the relationship between body mass index, cervical length and the risk of spontaneous preterm birth.

**Method:**

In this prospective cohort study, pregnant women between 16 and 24 weeks who fulfilled the criteria were recruited. Women with history of preterm birth were excluded. Demographic and clinical data (age, BMI, ethnicity, education level and parity) were obtained. Cervical length was measured using transvaginal scan. Patients were then followed up till delivery to determine their delivery gestation and outcome of delivery.

**Results:**

Out of 153 women who participated in this study, 146 women had cervical length of more than 30 mm, six had cervical length between 25 mm and 30 mm and one had cervical length of 24 mm. There were nine (9) cases of sPTB, with all of them being late preterm with normal midtrimester cervical length. Almost half of them (44%) were overweight/obese. A significant association was found between age, cervical length, and parity compared to BMI. Nevertheless, no significant association was seen between the BMI and risk of sPTB.

**Conclusion:**

This study demonstrates a higher BMI is associated with longer cervical length, but it is not necessarily protective against sPTB. Hence, we concluded there is a limited role in cervical length screening among low-risk women regardless of their BMI in predicting sPTB.

## Background

Preterm birth (PTB) rates have fluctuated for the past ten years, with more than one million children dying each year due to its complications [1]. Nearly 15% of all deliveries in Malaysia are contributed by PTB [2, 3]. The Malaysia National Neonatal Registry reported that out of 280,764 live births, about 3060 (24.5%) were premature (< 32 weeks), and 3415 (27.3%) were less than 1500 g birthweight [3]. Early detection and management of women who are at risk is essential to reduce the occurrence of sPTB and its related complications. Many survivors of PTB encountered an increased risk for neonatal health complications and long-term disabilities such as mental retardation, lung problems and cerebral palsy [4]. In addition, it was thought to put more financial, medical and emotional stress on the affected communities other than the perinatal issues [5, 6].

The majority of PTB remains unexplained. Even though methods have been taken to prevent preterm delivery, the incidence remained the same. Several factors were speculated to initiate PTB, including mechanical factors (such as uterine overdistension), inflammation with or without infection, circulatory disorders in cases like uteroplacental insufficiency, or a combination of several factors [7]. About two-thirds of all preterm births are spontaneous, while the rest are iatrogenic, for maternal or fetal indications [8]. Factors contributing to PTB include maternal factors such as pre-pregnancy body mass index (BMI), inter-pregnancy interval and psychosocial factors, obstetrics history such as previous PTB and medical disorders and pregnancy characteristics such as infections, multiple gestation, shortened cervix due to previous cervical surgery, antepartum hemorrhage and abnormal liquor volume [9, 10]. Most of these risk factors have a low predictive value, except for a history of previous PTB. Thus, the cause of spontaneous preterm labor (sPTB) remains unidentified in nearly half of all cases [11].

A past obstetric history of having preterm delivery is one of the most consistently reported risk factors for preterm delivery. A systematic review concluded that the absolute risk of recurrent sPTB was 25% (95% CI 24–26) after a previous preterm singleton delivery (risk of 57%, 95% CI 42–62) [12, 13]. Short cervical length, if found between 16 and 24 weeks, is an established risk factor for spontaneous PTB [14, 15]. The risk is inversely proportional to the cervical length [14, 15]. The International Federation of Gynaecology & Obstetrics (FIGO) 2015 recommended universal cervical length screening for predicting and preventing spontaneous preterm birth [16, 17]. A cut-off value of < 25 mm has been accepted worldwide, including in Malaysia, as having a high risk of spontaneous preterm labour. Nevertheless, cervical length (CL) tends to vary by race, parity and BMI, and can influence different populations differently [18].

There was limited data on whether the cut-off value of cervical length differs in different groups of women with different BMIs. Past studies found contradictory results regarding the association between BMI and risk of cervical length shortening, and PTB. Several studies found that women with lower BMI have a higher risk of PTB in contrast to obese women [19–21]. The data regarding the risk of sPTB in overweight and obese women is conflicting; some studies showed an increased risk of sPTB, while other studies showed that overweight and obese women have a lower risk of sPTB compared to normal-weight women [22].

A secondary analysis by Venkatesh et al. in 2020 found that overweight and obese women were more likely to have a longer cervical length compared to normal-weight women, but this does not necessarily protect them from PTB [21]. Han et al. in 2011 found a contradictory result where obese women have a higher risk of PTB. Despite this, the mechanism was not associated with a short cervix compared to underweight women [23, 24]. On the other hand, women with a low BMI have an odd ratio (OR) of 1.3 (95% CI 1.2–1.3) for sPTB [13]. The low BMI can be related to chronic malnutrition and nutritional deficiencies such as iron or zinc, which can negatively interfere with both fetal birth weight and immune system development [25]. Obese women were also found to have a higher risk for sPTB, with an increment of OR with an increase in BMI [25]. A study by Palatnik et al., 2017 showed that higher BMI is associated with longer mid-trimester cervical length and reduced risk for spontaneous PTB [15]. However, the decreased risk of spontaneous PTB may not be only associated with a longer cervical length. The reason for the potential protective effect of prematurity is unknown, and its mechanisms require further investigation. Due to this knowledge gap, we propose to look at the relationship between BMI, cervical length and risk of sPTB.

## Materials and methods

A prospective cohort study was conducted between 1st June 2021 and 28th February 2023 among antenatal women attending the antenatal clinic at either Hospital Pengajar Universiti Putra Malaysia or Hospital Serdang in Selangor, Malaysia. All patients who met the inclusion criteria; singleton pregnancy between 16 and 24 weeks’ gestation and no major fetal abnormality, were invited to participate in this study. Women with previous sPTB were excluded. Gestational age was determined using the last menstrual period and dating scan. Weight and height during antenatal booking in the first trimester were obtained from the participants’ antenatal book and used to calculate BMI. Three sonographers who had received the Fetal Medicine Foundation Certificate of Competence in cervical assessment performed the cervical length measurement. The cervical length was taken with an empty bladder via transvaginal ultrasound scan three times for each patient, and the average measurement was obtained in milimetres.

The sample size was calculated using formula *N* = p_0_q_0_ [Z(1-α/2) + Z(1-β) (√(p_1_q_1_/p_0_q_0_)]^2^ / (p_1_-p_0_)^2^, where n = sample size, 𝑍(1−𝛼/2): level of confidence = 95%. Hence, 𝑍(1−𝛼/2), level of confidence of 95% = 1.96, *z(1-β) = power of study (for a power of 80%, z = 0.84)*, p1 = 0.05 and p_2_ = 0.13. The calculated sample size was 122 after adding the dropout rate of 10%. This research was performed in accordance with the Declaration of Helsinki and approved by the Medical Research & Ethics Committee Ministry of Health Malaysia, ethical number NMRR-21-546-58894 on 12/05/2021 and Ethics Committee for Research Involving Human Subject of Universiti Putra Malaysia, ethical number JKEUPM-2021-153 on 31/05/2021. This study was registered under ClinicalTrials.gov on 08/06/2021 with the registration number NCT04922671. Pregnant women who fulfilled the criteria were recruited. Once they agreed, informed consent was obtained, and their information was collected from their antenatal book and hospital records. Data collected include age, weight, height and BMI at booking, parity, ethnicity, education level, occupation, last menstrual period, estimated delivery date, gestation age during transvaginal scanning, cervical length and co-morbidities. Following delivery, data on their gestation at delivery, mode of delivery and fetal outcomes were traced. Women who underwent iatrogenic preterm delivery were considered as dropouts.

Data was analysed using the IBM Statistical Package for Social Science (SPSS) version 27.0. Descriptive statistics were used to analyse demographic data. Fisher’s Exact test was used for associations between the categorical variables while Kruskal-Wallis test was used to determine the association between maternal characteristics (age, cervical length, ethnicity, education and parity) and BMI. Logistic regression models were used to compare cervical length and BMI.

## Results

**Fig. 1:**
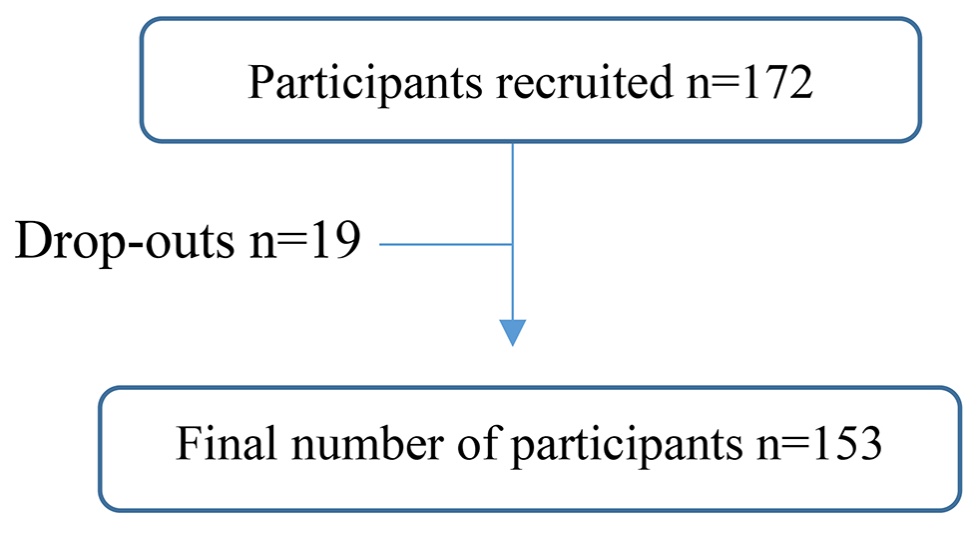
Number of participants

A total of 172 pregnant women were recruited. However, there were 19 dropouts (iatrogenic preterm delivery or missing delivery data), which left us with 153 participants, as shown in Fig. 1. Table 1 shows the demographic characteristics of the participants. Most participants were between 25 and 35 years old, with a mean age of 31.57 ± 4.68. Most of the participants were Malay (88.2%), followed by Chinese (5.3%) and Indians (6.5%). Meanwhile, the majority of the participants had a tertiary educational level (75.8%), followed by secondary (22.9%) and primary (1.3%). Among the 153 women, the mean booking BMI was 26.2 kg/m2 (standard deviation, SD 5.60). Up to 51% of participants were classified under the overweight and obese category, followed by the normal and underweight groups. Most of the participants were multipara, consisting of 56.2%, while 43.8% were nullipara.

Out of 153 women who participated in this study, 146 women had cervical length of more than 30 mm, six had cervical length between 25 mm and 30 mm and one had cervical length of 24 mm. None of these women received any intervention for the prevention of preterm birth.

**Table 1:** Demographic Characteristics of Participants

Variables	*N* (%) = 153 (100)
**Maternal Age**	
< 25 years old 25–34 years old ≥ 35 years old	7 (4.6) 103 (67.3) 43 (28.1)
**Ethnicity**	
Malay Chinese Indian	135 (88.2) 8 (5.3) 10 (6.5)
**Educational Level**	
Primary Secondary Tertiary	2 (1.3) 35 (22.9) 116 (75.8)
**BMI Class**	
Underweight Normal Overweight, Pre-obese and Obese	7 (4.6) 68 (44.4) 78 (51.0)
**Parity**	
Nulliparous Multiparous	67 (43.8) 86 (56.2)

Table 2 presents the baseline demographic and clinical data (age and cervical length) comparing women in three different groups of BMI using the World Health Organization (WHO) criteria. Group 1: Underweight (< 18.5 kg/m2), Group 2: normal (18.5 – <25 kg/m2) and Group 3: both overweight (25 – <30 kg/m2) and obese (≥ 30 kg/m2) [26]. There was significant association between age and cervical length with BMI. Older women were more likely to be overweight and obese. These group of women were more likely to have a longer cervical length compared to the underweight and normal weight women.

**Table 2:** Association between and cervical length with BMI

Maternal Characteristics	Overall *n* = 153 (100%)	Underweight (BMI < 18.5 kg/ m^2^) *n* = 7	Normal (BMI 18.5–24.9 kg/ m^2^) *n* = 68	Overweight & Obese, (BMI ≥25 kg/m^2^) *n* = 78	*P*-value
*Mean age (± SD) years	31.57 ± 4.68	31.43 ± 4.31	30.32 ± 4.52	32.67 ± 4.66	**0.017**
*Mean cervical length (± SD) mm	43.2 ± 7.50	36.0 ± 7.65	44.0 ± 8.02	43.0 ± 6.82	**0.05**

*Kruskal Wallis

Nevertheless, no significant association was seen between ethnicity and education level compared with BMI as shown in Table 3. Out of 153 women, there were nine cases of sPTB in which all were late preterm (35–36 weeks). None of these women have cervical length less than 25 mm, one woman has cervical length between 25 and 30 mm. Out of the 9 sPTB, 4 women (44%) belongs to overweight/obese group while the other 5 women (56%) belongs to normal BMI group.

**Table 3:** Association between demographic characteristics and BMI

Demographic Characteristics	Overall *n* = 153 (100%)	Underweight (BMI < 18.5 kg/ m^2^) *n* = 7	Normal (BMI 18.5–24.9 kg/ m^2^) *n* = 68	Overweight & Obese, (BMI ≥25 kg/m^2^) *n* = 78	*P*-value
**Ethnicity					
Malay Chinese Indian	140 (88.2) 6 (5.2) 7 (6.5)	7 (100.0) 0 (0) 0 (0)	59 (86.8) 3 (4.4) 6 (8.8)	74 (94.9) 3 (3.8) 1 (1.3)	0.256
**Educational Level					
Primary Secondary Tertiary	2 (1.3) 35 (22.9) 116 (75.8)	0 (0) 3 (42.9) 4 (57.1)	1 (1.5) 16 (23.5) 51 (75.0)	1 (1.3) 16 (20.5) 61 (78.2)	0.607
**Parity					
Nulliparous Multiparous	67 (43.8) 86 (56.2)	0 (0) 7 (100.0)	37 (54.4) 31 (45.6)	30 (38.5) 48 (61.5)	**0.012**

**Fisher’s exact test

The mean cervical length was 31.57 mm (SD 4.68). In univariate analysis using Pearson correlation analysis, no association was seen between the cervical length and gestation at delivery as shown on Table 4.

**Table 4:** Association between Cervical Length and Gestation at Delivery

Variables	Mean ± SD (range)	*p*-value
Cervical Length	31.57 ± 4.68	0.237
Gestation at Delivery	40.10 ± 24.27	

Majority of the participants delivered at term (94.1%) with only a minority of delivered preterm (5.9%). Table 5 showed that the mean midtrimester cervical length was similar between women who delivered at term and preterm among low-risk women. Univariate analysis using Pearson correlation analysis found no association between the mean cervical length of women who delivered at term or preterm.

**Table 5:** Mean Cervical length to Term/Preterm delivery

	Mean cervical length (mm)				*p*-value
	Term (*n* = 144)		Preterm (*n* = 9)		
	Mean	SD	Mean	SD	
Mean cervical length	43.2	7.5	43.1	8.4	0.935

**Fig. 2:**
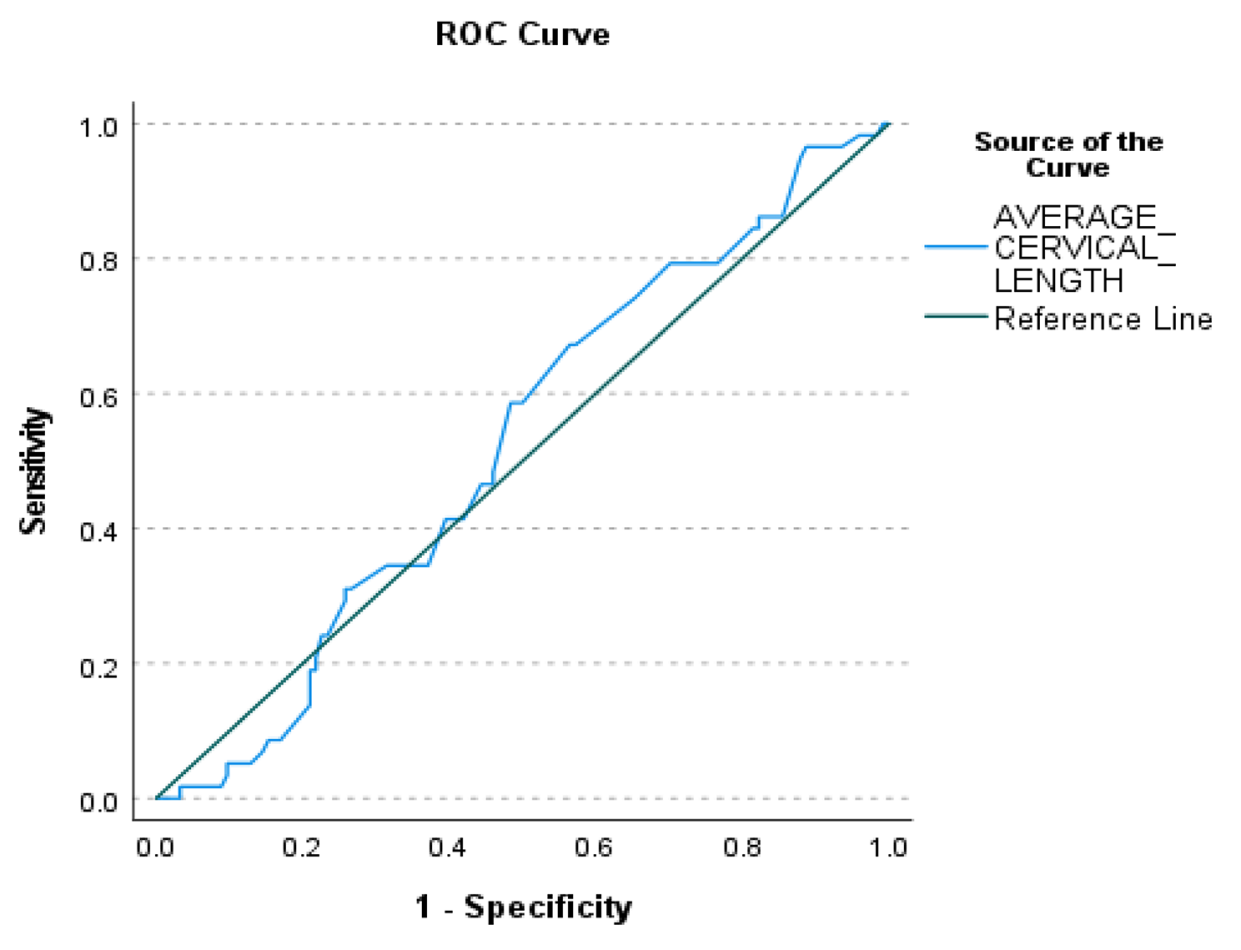
ROC Curve between Average Cervical Length and BMI

Figure 2 shows the receiver operating characteristic (ROC) curve between average cervical length and BMI. The best cut-off value among the participants was 39.5 mm with a sensitivity of 0.793 and 1-specificity of 0.702. The Area Under Curve (AUC) is 0.525.

## Discussion

This study found that most of the participants were Malay (91.5%) as the areas covered by these two hospitals resided by mostly Malay ethnic group, which is also the ethnic majority in Malaysia. Since the area is situated in the urban area, this explains the reason why nearly two-thirds (75.8%) of the participants have had tertiary educational level. Unsurprisingly, most of the participants (51%) were classified under the overweight and obese category. This correlates with the prevalence of obesity in Malaysia, which found that women are predisposed to obesity compared to men [27]. Several factors that may have contributed to the risk of obesity among women in this country include a sedentary lifestyle, occupation and unhealthy diet. Women with tertiary education are more likely to have an office job than someone who is less educated and usually involved in physically demanding work.

Meanwhile, we found a significant association between age, parity and cervical length by BMI. As the woman gets older and pregnant again, they tend to have a higher BMI and longer cervical length. It is common for women to retain weight postpartum and unable to regain their pre-pregnancy weight before subsequent pregnancy. During the postpartum period, women were more likely to eat high-saturated fatty acid food and become physically less active, thus contributing to weight retention [28, 29]. Interestingly, adequate sleep was protective for weight retention during postpartum [28]. Women are prone to insufficient sleep because they must care for their new baby. Hence, this may have resulted in postpartum weight retention. For the low-risk group of women (those without a history of sPTB), the increase in BMI with age and parity may have a protective effect as the cervical length may be longer compared to the younger and nullipara women.

Nevertheless, no significant association between education level and BMI was seen. A study among Brazilian mothers found a higher weight loss postpartum among educated mothers, which contradicts our finding [30]. This difference could be due to different confinement practices whereby Malaysian mothers were restricted with their cultures and traditions despite having a higher education level. Our ancestors much influenced our practices. On the other hand, there was a lack of previous studies that looked specifically at different Malaysian ethnicities and postpartum weight retention. We found no significant association for this.

Our analysis found no significant association between BMI, cervical length and risk of sPTB. These findings disagree with the findings by Granese et al., which was conducted among the Italian population, who found that maternal BMI has a significant association with the risk of having preterm birth [31]. The differences could be due to the study being conducted retrospectively, women with previous PTB were included, and many other confounding risk factors could have contributed to the risk of sPTB, such as vaginal or urinary tract infections and maternal medical disorders. As our study only includes low-risk women, only a minority of cases delivered preterm, largely late preterm. Late preterm birth is less likely to occur due to cervical incompetence, which usually presents with short cervical length. It could be induced by other factors such as maternal infection or medical background. Our findings suggest that a universal screening of cervical length among low-risk women may not be beneficial and play a limited role in predicting sPTB.

Venkatesh et al. suggested that maternal obesity is associated with sPTB, although their cervical length in the early part of pregnancy may be longer than their normal BMI counterparts [21]. The reasons for this could be influenced by different physiology, inflammatory reactions, amount of fat and soft tissues in the pelvis and medical backgrounds [32]. Their study, however, established no significant association between cervical length and gestation at delivery, which is a similar finding to our study. A longer mid-trimester cervical length did not necessarily indicate a longer gestation period. However, this may not be the case among high-risk women who experience previous sPTB. Other factors that may play a role in contributing to sPTB among low-risk women include medical, social, genetic and environmental factors that could influence the timing of delivery. With a similar mean mid-trimester cervical length as demonstrated in Table 5, one should not focus on cervical length alone. Screening on common medical disorders contributing to sPTB, asking questions looking for possible urine or vaginal infections, and screening for infections should routinely continue to identify low-risk women at risk of sPTB. Our regression analysis shows that low-risk women with cervical lengths of more than 39.5 mm at mid-trimester are more likely to deliver at term. However, as the area under the curve is nearly 0.5, cervical length and BMI alone may not be a good predictor in low-risk women, in particular those without previous sPTB.

Our study has several strengths. Being a prospective design, this reduces bias and confounders. The accuracy of cervical length measurement is safeguarded as only trained personnel performing the measurements were done repeatedly by taking the average. Limitations include the small number of samples, which results in a low number of sPTB. Secondly, this study may only be able to represent part of the community in Malaysia as the participants were mainly from the urban areas with higher education status. Therefore, it may not be generalised to a larger population.

## Conclusion

There was no significant association between BMI, cervical length and risk of sPTB among low-risk women. Hence, universal cervical length screening alone may have a limited role in predicting sPTB among low-risk women. However, universal screening among nullipara may be considered in reducing the rate of sPTB and improving the care among at-risk women.

## Data Availability

The datasets used and/or analysed during the current study available from the corresponding author on reasonable request.
